# Identification of Heparan Sulfate in Dilated Cardiomyopathy by Integrated Bioinformatics Analysis

**DOI:** 10.3389/fcvm.2022.900428

**Published:** 2022-05-27

**Authors:** Wenyu Song, Fujian Lu, Zequan Ding, Liqi Huang, Kui Hu, Jinmiao Chen, Lai Wei

**Affiliations:** ^1^Department of Cardiovascular Surgery, Zhongshan Hospital, Fudan University, Shanghai, China; ^2^Department of Cardiology, Boston Children's Hospital and Harvard Medical School, Boston, MA, United States; ^3^Department of Pediatric Surgery, Children's Hospital of Nanjing Medical University, Nanjing, China; ^4^Department of Cardiovascular Surgery, Guizhou Provincial People's Hospital, Guiyang, China

**Keywords:** heparan sulfate, cardiac inflammation and fibrosis, bioinformatics, molecular subtype, dilated cardiomyopathy

## Abstract

**Objectives:**

Heparan sulfate (HS) forms heparan sulfate proteoglycans (HSPGs), such as syndecans (SDCs) and glypicans (GPCs), to perform biological processes in the mammals. This study aimed to explore the role of HS in dilated cardiomyopathy (DCM).

**Methods:**

Two high throughput RNA sequencing, two microarrays, and one single-cell RNA sequencing dataset of DCM hearts were downloaded from the Gene Expression Omnibus (GEO) database and integrated for bioinformatics analyses. Differential analysis, pathway enrichment, immunocytes infiltration, subtype identification, and single-cell RNA sequencing analysis were used in this study.

**Results:**

The expression level of most HSPGs was significantly upregulated in DCM and was closely associated with immune activation, cardiac fibrosis, and heart failure. Syndecan2 (SDC2) was highly associated with collagen I and collagen III in cardiac fibroblasts of DCM hearts. HS biosynthetic pathway was activated, while the only enzyme to hydrolyze HS was downregulated. Based on the expression of HSPGs, patients with DCM were classified into three molecular subtypes, i.e., C1, C2, and C3. Cardiac fibrosis and heart failure were more severe in the C1 subtype.

**Conclusion:**

Heparan sulfate is closely associated with immune activation, cardiac fibrosis, and heart failure in DCM. A novel molecular classification of patients with DCM is established based on HSPGs.

## Introduction

Dilated cardiomyopathy (DCM) is a kind of cardiomyopathy characterized by dilatation of ventricular cavities and impaired systolic function. This cardiovascular disorder is closely associated with progressive heart failure and even sudden cardiac death. The pathogenesis of DCM is a complicated process with high heterogeneity (1). Notably, cardiac inflammation and fibrosis participate in the pathological process of DCM (2). However, the molecular mechanism of this process is still unclear.

Heparan sulfate (HS) is a linear polysaccharide widely expressed on the cell membrane and in the extracellular matrix. It participates in large scale of biological processes that include cell-cell adhesion and transduction of intracellular signaling pathways (3). HS functions through binding to different core proteins and forming various kinds of heparan sulfate proteoglycans (HSPGs). HSPGs include syndecans (SDC1–4), glypicans (GPC1–6) on the cell membrane and HSPG2, agrin (AGRN), and COL18A1 in the extracellular matrix (3). Over the decades, certain HSPGs, such as SDC1 and SDC4, have been identified to be associated with worse cardiac function and poor prognosis of DCM (4, 5). However, these studies did not provide an overview of HS and its underlying mechanism. Since HS participates in the component of the extracellular matrix and is widely expressed in cardiac fibroblasts (2), it is valuable to investigate the role of HS on cardiac fibrosis in DCM.

In this study, we integrated high throughput sequencing, microarray, and single-cell RNA sequencing datasets to illustrate the role of HS in DCM. We further explored a molecular classification of DCM based on HS heterogeneity among patients with DCM.

## Materials and Methods

### Data Acquisition and Preprocessing

Raw data of DCM and normal heart tissues were acquired from the Gene Expression Omnibus (GEO) database (https://www.ncbi.nlm.nih.gov/geo/), including GSE116250 (6), GSE141910, GSE42955 (7), GSE5406 (8), and GSE121893 (9). The details of the transcriptome datasets are shown in Supplementary Table S1. The downloaded data underwent a probe-gene symbol transformation with Perl (strawberry-Perl-5.32.1.1, https://strawberryperl.com/) according to their platform files. The high throughput RNA sequencing datasets were used as the training data. These two datasets were batched and normalized to eliminate the batch effects caused by different experiments and platforms through R package “sva” (version 3.42.0) (10). R function “ComBat” was used to eliminate the batch effects and R functions “rbind” and “normalizeBetweenArrays” were conducted to bind and normalize these datasets. The method of normalization was set as default. Microarray datasets were used as the validation datasets and underwent similar preprocessing.

### Principal Component Analysis (PCA)

The pre- and post-batch-normalized data were tested by sample clustering analysis *via* PCA (11). R function “prcomp” was used to perform PCA. Dot plots were presented by R package “ggplot2” (version 3.3.5).

### Evaluation of Immunocytes Infiltration

In this study, immunocytes were calculated *via* two independent methods, CIBERSORT (12) and microenvironment cell population-counter (MCPcounter) (13). LM22 (http://CIBERSORT.stanford.edu/) was applied as a reference expression signature with 100 permutations. The immunocytes were calculated by R function “CIBERSORT.” The immunocytes were also estimated with R function “MCPcounter.estimate” in R package “MCPcounter” (version 1.2.0). The results were visualized using R packages “ggplot” and “ggpubr” (version 0.4.0).

### Identification of Differential Expressed Genes (DEGs)

After a distribution analysis, the expression of most genes was non-normally distributed. Thus, differential analysis of the transcriptome data and immunocytes between groups was conducted based on a non-parametric test using the R function “pairwise.wilcox.test” under a Bonferroni method. The expression levels of HS genes in DCM and normal heart were shown in the heatmap using the R function “pheatmap.”

### Pathway Enrichment Analysis

In this study, the Kyoto Encyclopedia of Genes and Genomes (KEGG) pathway gmt file was downloaded from gene set enrichment analysis (GSEA, http://www.gsea-msigdb.org/gsea/index.jsp). This file was further used for gene set variation analysis (GSVA) to calculate the enrichment score of pathways with R package “GSVA” (version 1.42.0) and “GSEABase” (version 1.56.0) (14). After a distribution analysis, the enrichment score of most pathways was non-normally distributed. Then the differential analysis of the enrichment score was conducted with the R function “pairwise.wilcox.test” under a Bonferroni method according to the group division. The enrichment score was shown in the bar plot and heatmap.

### Identification of DCM Subtypes

Heparan sulfate proteoglycans were chosen as candidate genes to identify the DCM subgroup through a non-negative matrix factorization (NMF) clustering using the R package “NMF” (version 0.23.0) (15). The value of k where the magnitude of the cophenetic correlation coefficient began to fall at a steep amplitude was taken as the optimal number of clusters. R function “consensusmap” was used to plot the heatmap of clustering with the k value from 2 to 10. R function “pairwise.wilcox.test” was applied to compare the index of cardiac fibrosis and heart failure between multiple subclusters using a Bonferroni method.

### Screening of Hub HSPGs, KEGG Pathways, and Immunocytes

Two machine learning algorithms were applied to screen hub HSPGs genes and immunocytes that include least absolute shrinkage and selection operator (LASSO) regression (16) and support vector machine recursive feature elimination (SVM-RFE) (17). R package “glmnet” (version 4.1-2) was used to conduct LASSO regression. R packages “e1071” (version 1.7-9), “kernlab” (version 0.9-29), and “caret” (version 6.0-90) were applied to conduct SVM-RFE algorithm. Hub genes were defined as the intersection of DEGs selected by LASSO and SVM-RFE.

### Single-Cell RNA Sequencing Analysis

GSE121893 was preprocessed and analyzed using the R package “Seurat” (version 4.0.5) (18). This dataset contains heart specimens with coronary heart disease (*n* = 2), DCM (*n* = 3), and normal heart (*n* = 2). The cardiac fibroblasts from DCM patients were selected due to the cell annotation in the supplementary files of the original draft (9). Analysis of single-cell clustering and cell trajectory was conducted with R packages “Seurat”, “monocle” (version 2.22.0), and “celldex” (version 1.4.0).

### Correlation Analysis

The correlation analyses between genes, immunocytes, and pathways were conducted with the R function “cor.test” using Spearman's method. The results of correlation analyses were shown with the R package “corrplot” (version 0.90), “ggplot2” and “igraph” (version 1.2.7).

### Protein-Protein Interaction (PPI) Network

Protein-protein interaction network of HSPGs was downloaded from the STRING database (https://www.string-db.org/) and visualized by Cytoscape (version 3.9.1).

## Results

### HSPGs and Cardiac Fibrosis and Heart Failure in DCM

**Figure 1:**
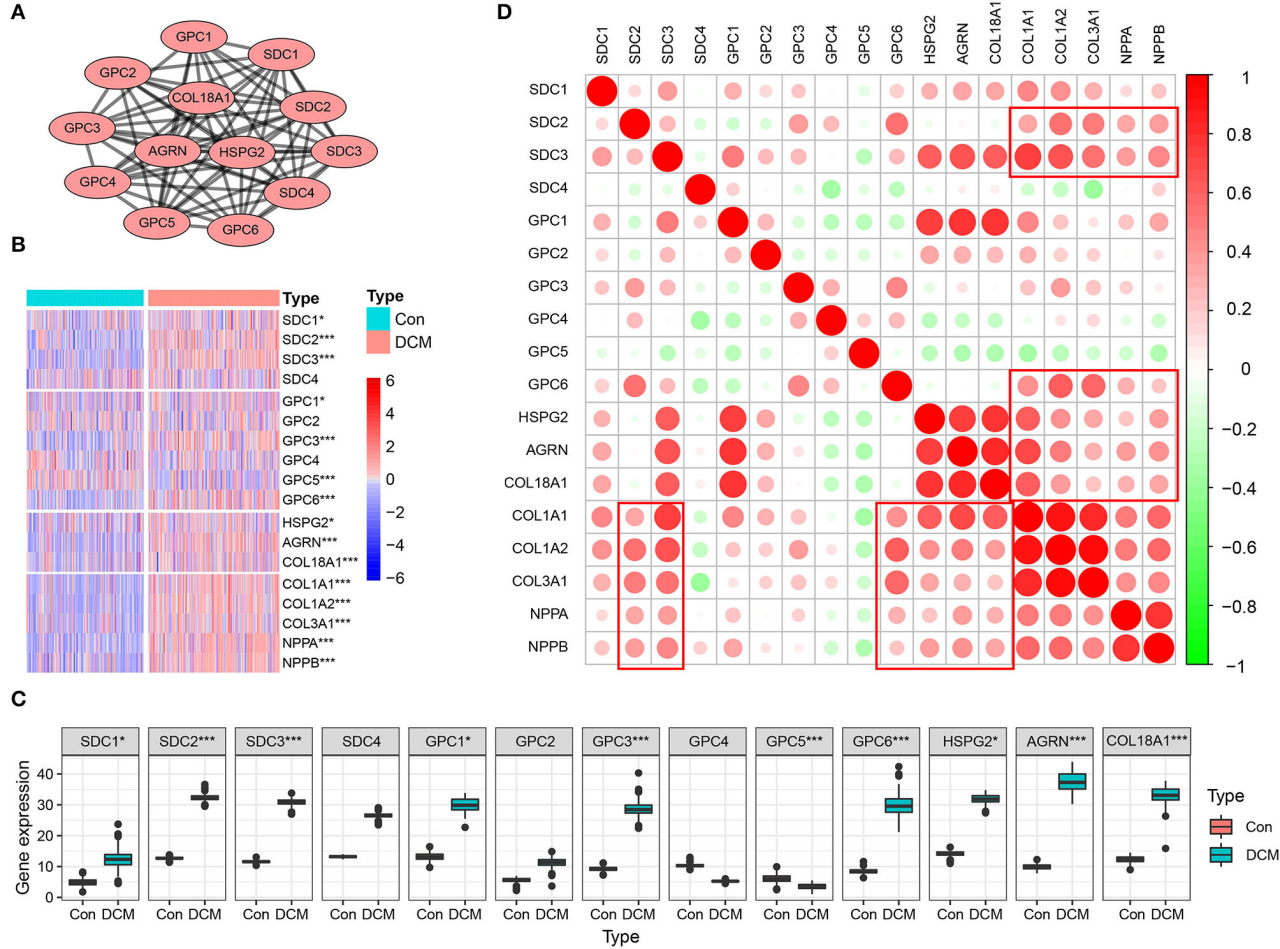
Heparan sulfate proteoglycans (HSPGs) and cardiac fibrosis and heart failure in dilated cardiomyopathy (DCM). **(A)** Protein-protein interaction (PPI) network of HSPGs. Each node represents a type of HSPG, and edges connect the interacted HSPGs. **(B)** Heatmap of HSPGs, cardiac fibrosis, and heart failure indexes in DCM and normal specimens. Red represents higher expression and blue represents lower expression, respectively. **(C)** Expression abundance of HSPGs. **(D)** Correlation between HSPGs, cardiac fibrosis and heart failure. Red represents higher correlation and green represents lower correlation, respectively. *p < 0.05, ***p < 0.001.

Multiple datasets were combined and normalized to eliminate batch effects (Supplementary Figures S1A–D). Previous reported HSPGs were extracted and shown in a PPI network (Figure 1A). *Via* a differential analysis, we found that the enrichment score of HSPGs in DCM was significantly upregulated both in the training and validation datasets (Supplementary Figures S1E,F). Specifically, the expression of most SDCs and GPCs was significantly upregulated in DCM when compared to normal heart (logFC > 1 and *p* < 0.05, Figure 1B and Table 1). GPC5 was the only downregulated HSPG in DCM with relatively low expression abundance (Figure 1C). The expressions of HS in the extracellular matrix were all significantly upregulated, such as HSPG2, AGRN, and COL18A1. The level of cardiac fibrosis and heart failure in DCM was significantly increased (logFC > 1 and *p* < 0.05, Figure 1B), which were indicated by collagen type I alpha 1 chain (COL1A1), collagen type I alpha 2 chain (COL1A2), collagen type III alpha 1 chain (COL3A1), natriuretic peptide A (NPPA), and natriuretic peptide B (NPPB). *Via* a correlation analysis, we found that SDC2, SDC3, GPC6, HSPG2, AGRN, and COL18A1 were statistically correlated with above five indexes (Figure 1D).

**Table 1:** LogFC and value of *p* of heparan sulfate proteoglycans (HSPGs), cardiac fibrosis, and heart failure indexes.

**Gene**	**logFC**	***p*-Value**
SDC1	1.27	<0.05^*^
SDC2	1.30	<0.001^***^
SDC3	1.36	<0.001^***^
SDC4	1.01	>0.05
GPC1	1.16	<0.05^*^
GPC2	1.00	>0.05
GPC3	1.53	<0.001^***^
GPC4	−0.97	>0.05
GPC5	−0.67	<0.001^***^
GPC6	1.69	<0.001^***^
HSPG2	1.15	<0.05^*^
ARGN	1.81	<0.001^***^
COL18A1	1.37	<0.001^***^
COL1A1	2.51	<0.001^***^
COL1A2	2.06	<0.001^***^
COL3A1	1.73	<0.001^***^
NPPA	5.32	<0.001^***^
NPPB	4.61	<0.001^***^

* *P < 0.05*,

*** *P < 0.001*.

### HSPGs in Cardiac Fibroblasts of DCM

**Figure 2:**
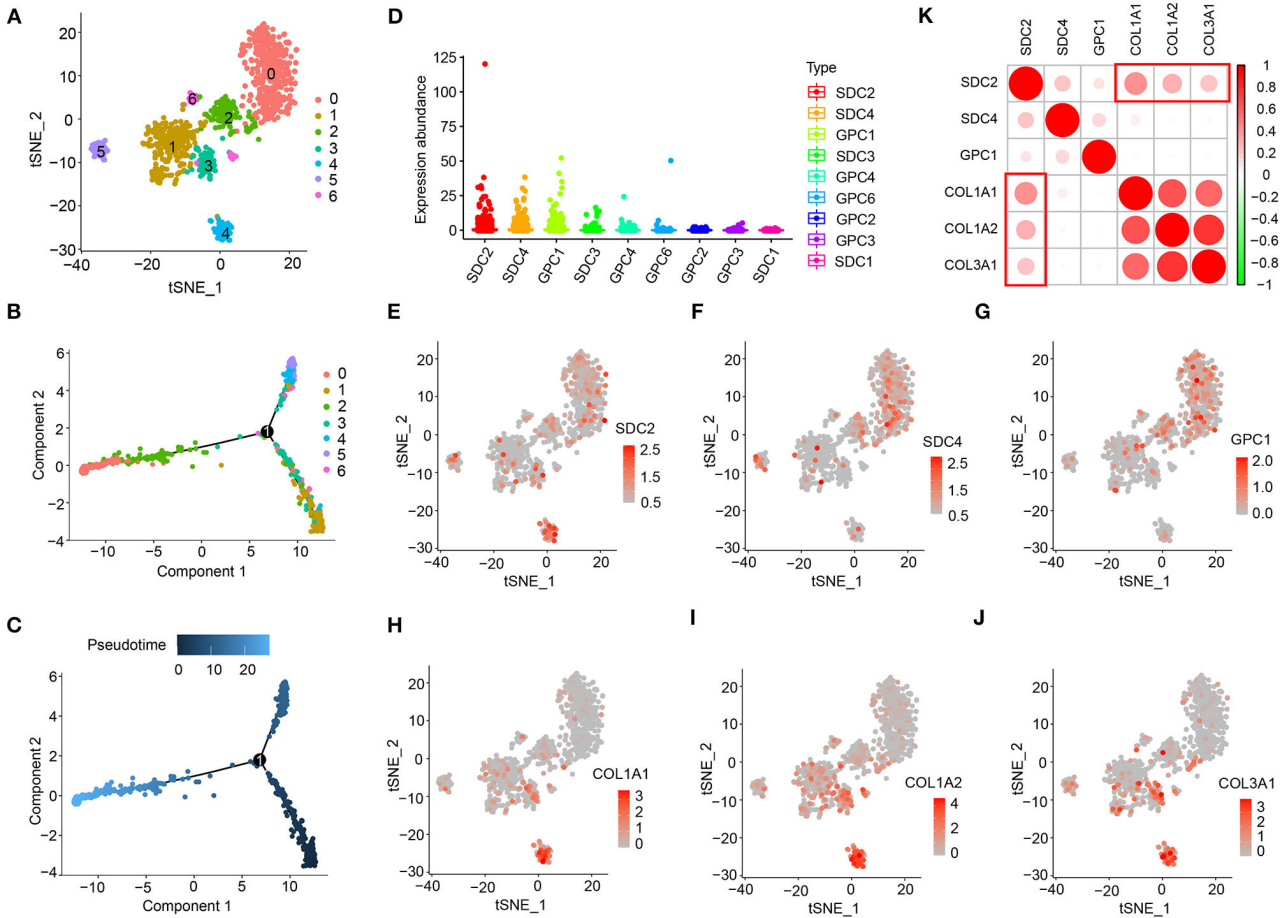
Heparan sulfate proteoglycans (HSPGs) in cardiac fibroblasts of dilated cardiomyopathy (DCM). **(A)** TSNE clustering. *X*- and *Y*-axis represent two dimensions after dimension reduction through t-distribution stochastic neighbor embedding (TSNE). **(B,C)** Cell trajectory. *X*- and *Y*-axis represent two components while calculating cell trajectories. **(D)** The expression abundance of cell membrane HSPGs. **(E–J)** The expression and distribution of SDC2, SDC4, GPC1, COL1A1, COL1A2, and COL3A1. *X*- and *Y*-axis represent two dimensions after dimension reduction through TSNE. Red represents a higher expression. (K) Correlation between SDC2, SDC4, GPC1, COL1A1, COL1A2, and COL3A1. Red represents higher correlation and green represents lower correlation, respectively.

To further understand the association between HSPGs and cardiac fibrosis, a single-cell RNA sequencing analysis of cardiac fibroblasts was performed. A total of 973 cardiac fibroblasts were classified into seven clusters, indicating the heterogeneity of fibroblasts in DCM (Figure 2A). The cell trajectory plot is shown in Figure 2B and C. The expression abundance of SDC2, SDC4, and GPC1 was among the top three (Figure 2D). By visualizing SDC2, SDC4, GPC1, COL1A1, COL1A2, and COL3A1, we found that SDC2 was highly correlated with the index of cardiac fibrosis when compared to SDC4 and GPC1 (Figures 2E–K). These results revealed that SDC2 may play a key role in the cardiac fibroblasts of DCM.

### Immunocyte Infiltration in DCM and Normal Hearts

**Figure 3:**
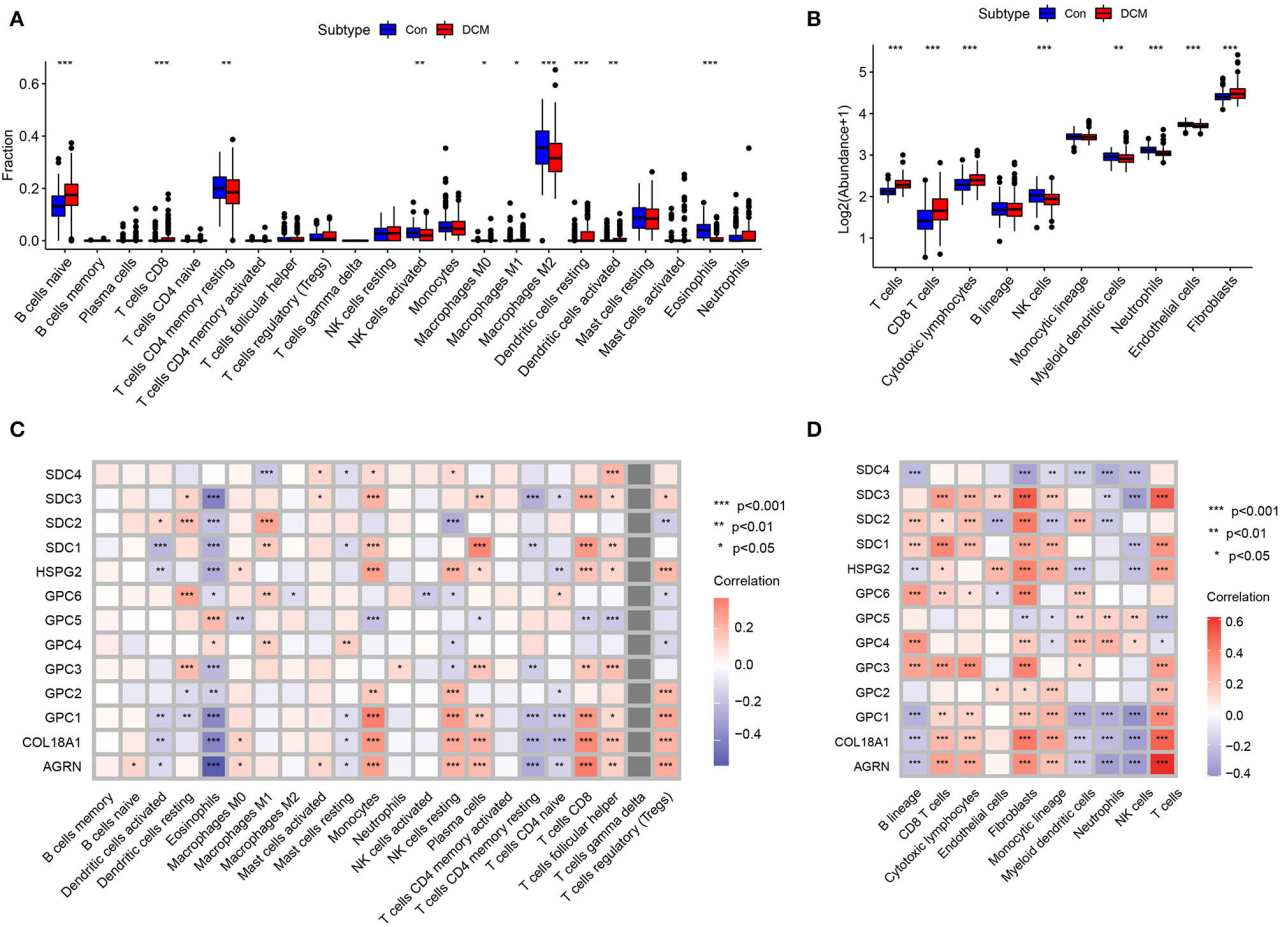
Immunocytes infiltration in dilated cardiomyopathy (DCM) and normal hearts. The composition and abundance of immunocytes were calculated by **(A)** CIBERSORT. **(B)** MCPcounter; Correlation between heparan sulfate proteoglycans (HSPGs) and immunocytes. **(C)** CIBERSORT. **(D)** MCPcounter. Red represents higher correlation and blue represents lower correlation, respectively. The name of immunocytes originated from official files and software of CIBERSORT and MCPcounter, *p < 0.05, **p < 0.01, ***p < 0.001.

Immunocyte infiltration is an important driver of cardiac fibrosis. To investigate whether HSPGs are associated with inflammation and the immune microenvironment in DCM, immunocyte infiltration was first calculated by two independent methods that include CIBERSORT and MCPcounter. For the CIBERSORT manner, the fractions of 22 kinds of immunocytes in DCM and normal heart are shown in Figure 3A. Naïve B cells, CD8^+^ T cells, M0 macrophages, M1 macrophages, and dendritic cells were significantly higher in DCM than in normal heart. Memory resting CD4^+^ T cells, activated NK cells, M2 macrophages, and eosinophils were downregulated in DCM. For the MCPcounter manner, the abundance of 10 kinds of immunocytes in DCM and normal heart is shown in Figure 3B. CD8^+^ T cells, cytotoxic lymphocytes, and fibroblasts were upregulated, while NK cells, myeloid dendritic cells, neutrophils, and endothelial cells were downregulated in DCM. *Via* a correlation analysis, we found that HSPGs were widely correlated with immunocytes in DCM (Figures 3C,D).

### HS Metabolic Genes and Immunocytes, Cardiac Fibrosis and Heart Failure in DCM

**Figure 4:**
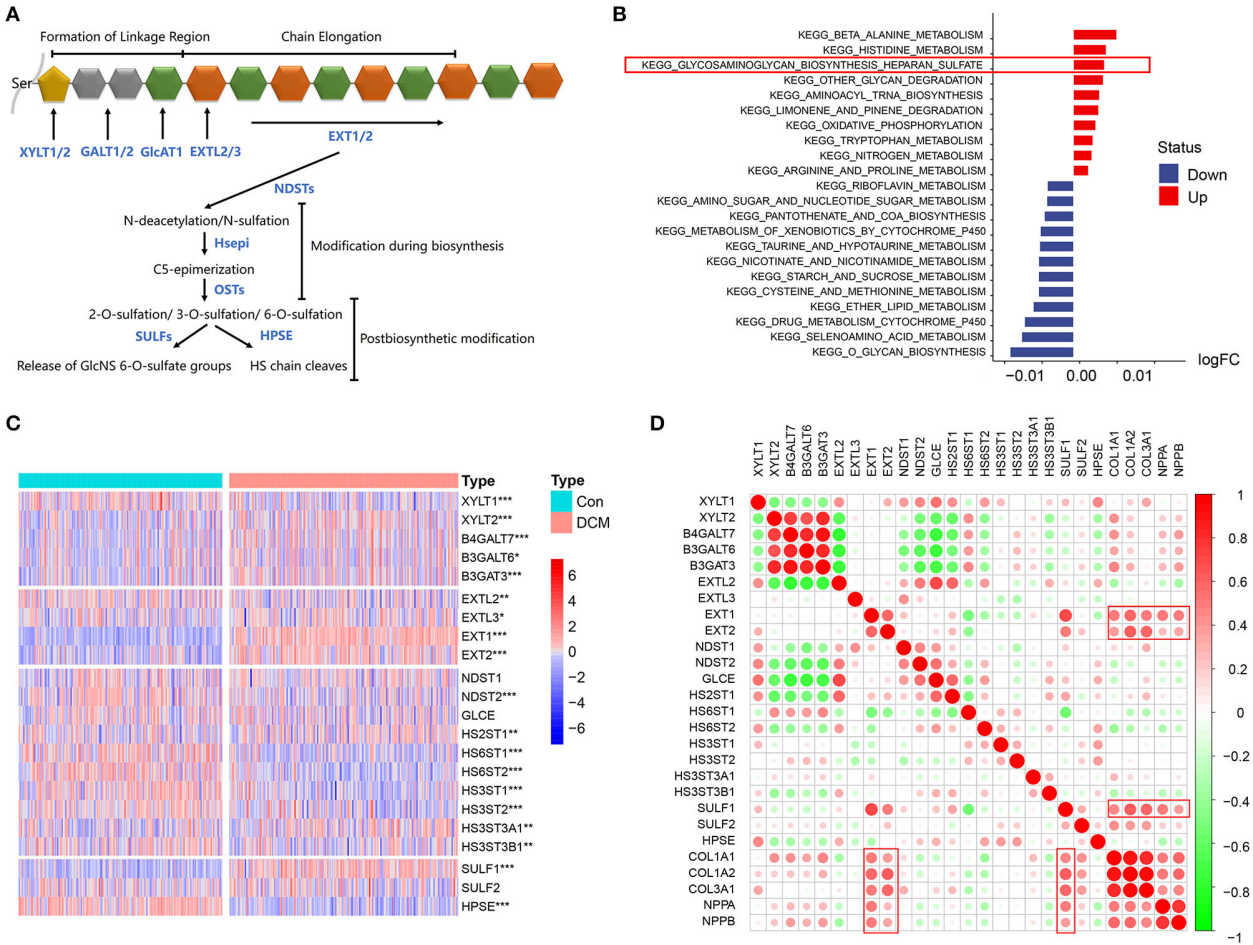
HS metabolic genes and immunocytes, cardiac fibrosis, and heart failure in dilated cardiomyopathy (DCM). **(A)** Process of HS metabolism. **(B)** Gene set variation analysis (GSVA) of KEGG pathways enriched in DCM. Red represents upregulated pathways and blue represents downregulated pathways, respectively. **(C)** Heatmap of HS metabolic genes in DCM and normal specimens. Red represents higher expression and blue represents lower expression, respectively. **(D)** Correlation between HS biosynthesis genes, cardiac fibrosis, and heart failure. Red represents higher correlation and green represents lower correlation, respectively. *p < 0.05, **p < 0.01, ***p < 0.001.

The mechanism of HSPG overexpression was investigated. First, the process of HS polysaccharide metabolism is visualized in Figure 4A. *Via* GSVA, we aimed to explore whether the HS metabolic pathway was altered in DCM. Significantly altered KEGG metabolic pathways are shown in Figure 4B. HS biosynthetic pathway was ranked third, which indicated that HS biosynthesis was remarkably activated in DCM. Available HS metabolic genes were obtained and are shown in Figure 4C. *Via* a correlation analysis, we found that three important enzymes that include Exostosin 1 (EXT1), Exostosin 2 (EXT2), and SULF1 to synthesize HS were significantly correlated with abovementioned cardiac fibrosis and heart failure indexes (*p* < 0.05, Figure 4D). Notably, the expression of heparanase (HPSE) was downregulated (logFC = −0.51, *p* < 0.05), which was the only enzyme to hydrolyze HS.

### Identification of DCM Subtypes Based on HSPGs

**Figure 5:**
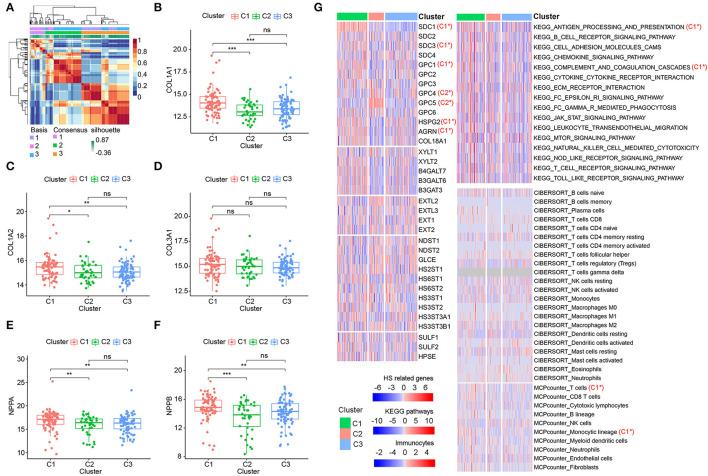
Identification of dilated cardiomyopathy (DCM) subtypes based on heparan sulfate proteoglycans (HSPGs). **(A)** NMF clustering heatmap when *k* = 3. **(B–D)** The expression of COL1A1, COL1A2, and COL3A1 among three identified subtypes. **(E,F)** The expressions of NPPA and NPPB among three identified subtypes. **(G)** Heatmap of HS-related genes, immune-associated pathways, and immunocytes in the subtypes of DCM. Red represents a higher expression/score and blue represents the opposite. C1* represents a significant higher expression in C1 subtype. C2* represents a significant higher expression in C2 subtype. The name of immunocytes originated from official files and software of CIBERSORT and MCPcounter. *p < 0.05, **p < 0.01, ***p < 0.001, ns = not significant.

Considering the importance of HSPGs in DCM, this set of genes was then applied to identify molecular subtypes using an NMF manner. NMF clustering heatmaps and cophenetic correlation coefficient plots are shown in Supplementary Figures S2, S3. According to the cophenetic correlation coefficients, k was chosen as 3 for the optimal number of clusters (Figure 5A). Then, 203 patients with DCM in the training dataset were clustered into three subtypes, i.e., C1 (*n* = 79), C2 (*n* = 40), and C3 (*n* = 84). Expression levels of COL1A1, COL1A2, NPPA, and NPPB were significantly higher in C1 than in C2 and C3 (Figures 5B–F). Furthermore, the clustering of DCM was validated using the microarray datasets (Supplementary Figures S4A–F). Similarly, cardiac fibrosis and heart failure were more severe in C1 subtype of the validation dataset. Subsequently, HS-related genes, immune-related pathways, and immunocytes in the subtypes of DCM were shown in the heatmap (Figure 5G). For HSPGs, the expressions of SDC1, SDC3, GPC1, HSPG2, and AGRN were significantly higher in C1 subtype than in C2 and C3 subtypes (*p* < 0.05). GPC4 and GPC5 were mainly expressed in C2 subtype. No significant alteration was observed in C3 subtype. For immune pathways, complement and coagulation cascades and antigen processing and presentation pathways were enriched in C1 subtype, while no immune pathway was enriched in C2 and C3 subtypes. For immunocytes, the abundance of T cells and monocytic lineage were higher in C1 subtype. No immunocytes were significantly infiltrated in C2 and C3 subtypes.

### Screening Hub HSPGs and Immunocytes in C1 Subtype

**Figure 6:**
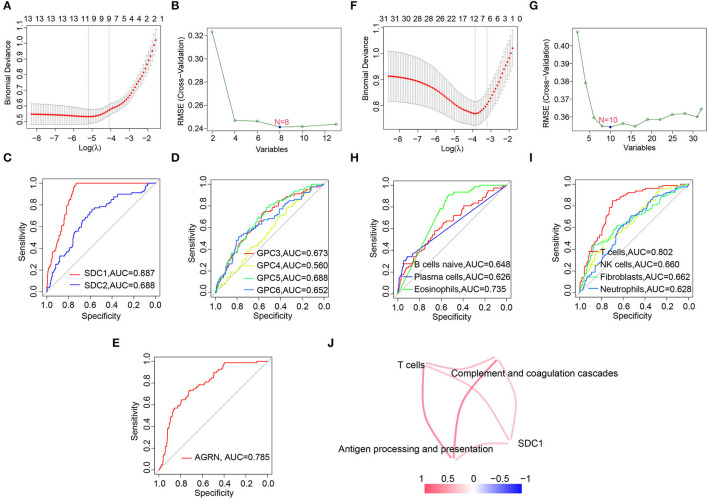
Screening hub heparan sulfate proteoglycans (HSPGs) and immunocytes in C1 subtype. **(A)** Least absolute shrinkage and selection operator (LASSO) regression. **(B)** SVM-RFE of screening HSPGs. **(C–E)** The area under the curve (AUC) curve of HSPGs. **(F)** LASSO regression. **(G)** Support vector machine recursive feature elimination (SVM-RFE) of screening immunocytes. **(H,I)** AUC curve of immunocytes. **(J)** Correlation network between SDC1, T cells, and certain Encyclopedia of Genes and Genomes (KEGG) immune-associated pathways and immunocytes. The name of immunocytes originated from official files and software of CIBERSORT and MCPcounter. Edge represents a correlation between two notes. Red represents higher correlation and blue represents lower correlation.

Since C1 subtype was identified as the most severe subtype of DCM, we screened the hub HSPGs and immunocytes in C1 subtype *via* LASSO regression and the SVM-RFE algorithm (Figures 6A,B,F–G). For the HSPGs, SDC1, SDC2, GPC3, GPC4, GPC5, GPC6, and AGRN were the intersection genes calculated by LASSO and SVM-RFE. The receiver operating characteristic (ROC) curves of these genes (Figures 6C–E) showed that SDC1 was the best HSPG for identifying C1 subtype [area under the curve (AUC) = 0.887]. For the immunocytes, naïve B cells, plasma cells, eosinophils (CIBERSORT), T cells, NK cells, neutrophils, and fibroblasts (MCPcounter) were the intersection immunocytes. The ROC curves of these immunocytes (Figures 6H,I) revealed that the abundance of T cells (MCPcounter) was the best immunocyte for identifying C1 subtype (AUC = 0.802). The predicted effectiveness of SDC1 and T cells was confirmed using the validation datasets (Supplementary Figures S4G,H). Further, the correlation networks between SDC1, T cells, and certain immune-associated KEGG pathways are shown in Figure 6J.

## Discussion

Heparan sulfate has been identified as an important regulator of cardiac inflammation and fibrosis. In this study, an overall upregulation of HS was found to be closely associated with immune activation, cardiac fibrosis, and heart failure in DCM. Moreover, we established a novel molecular classification of DCM based on HSPGs.

Heparan sulfate regulates intracellular signaling through binding to signal molecules with its polysaccharide chains. Certain fibrogenic pathways can be controlled through HS in tissue remodeling. For example, transforming growth factor beta (TGF-β) can induce fibrosis through binding to HS on the cell membrane of fibroblasts. On the contrary, inhibition of HS blocks TGF-β signaling pathway and alleviates fibrosis (19). In addition, HS is widely expressed in the extracellular matrix and serves as a critical component in fibrotic tissues. Consistently, our present study revealed overexpression of HS both on the cell membrane and in the extracellular matrix through analyses of DCM transcriptome datasets. Moreover, HS was closely associated with collagen expression of cardiac fibroblasts at the single-cell level. These results emphasized potential role of HS in cardiac fibrosis of DCM.

Although upregulation of HS was found in DCM, the potential mechanism was largely unclear. A previous study hypothesized that HS was induced in pressure overload hearts through transcription of core protein SDC4 (20). However, the expression of core proteins is mainly determined by the HS polysaccharide content (21). Here, our data showed that the HS polysaccharide biosynthesis KEGG pathway was ranked third among all altered pathways in DCM. HS biosynthesis is a complex biological process and EXTs and exostosin like glycosyltransferase (EXTLs) are key enzymes of this process (3). We found that significant upregulation of EXT1 and EXT2 was closely associated with cardiac fibrosis and heart failure in DCM, which partly explained the potential molecular mechanism of HS biosynthetic activation in DCM. Moreover, HPSE is the only enzyme to hydrolyze HS and was downregulated in DCM. In summary, we hypothesized that HS overexpression in DCM may be related to both the transcription of core proteins and the alteration of HS metabolism.

Heparan sulfate overexpression in DCM indicated a potential treatment by targeting HS. Currently, PI-88 (HS analog) has been already used to treat hepatocellular carcinoma and melanoma (22). In addition, our previous work reported an anti-diabetic effect by targeting HS. OGT2115 protected islet β cells against streptozotocin-induced inflammation and apoptosis in mice by regulating intra-islet HS (23). Moreover, inhibition of HS was reported to protect against fibrosis in kidney (24), lung (25), and cornea (26). Whether anti-fibrosis through targeting at HS is also effective in DCM deserves in-depth investigation.

In this study, we further found HS heterogeneity between patients with DCM and identified three molecular subtypes (C1, C2, and C3) based on HS. Cardiac fibrosis and heart failure were more severe in the C1 subtype, suggesting that these patients with DCM may have a poor prognosis. Thus, inhibition of HS may be a potential therapy, especially for C1 subtype of patients with DCM. Moreover, the characteristics of subtype C1 were further identified *via* LASSO regression and SVM-RFE. The expression of SDC1 and the abundance of T cells were screened as hub gene/immunocyte in C1 subtype. However, the prediction effectiveness of the validation dataset was not as high as the training data probably due to the insufficient sample capacity of microarray datasets.

This study has some limitations. First, it only revealed that HS-related genes were statistically associated with immune activation through bioinformatics analyses. The causal relationship needs further *in vivo* and *in vitro* experiments. Second, it is difficult to confirm these subtypes of DCM by endocardial biopsy in clinical practice. Thus, it required other ways to further confirm C1 patients, such as biomarkers in the peripheral blood. Last, DCM is highly heterogeneous, thus the molecular classification for DCM may vary among different pathobiology.

Despite the limitations, this study has some potential aspects for clinical translation. First, HS has been identified as a potential candidate for DCM biomarker through hundreds of specimens from existing datasets. This biomarker in peripheral blood may be an alternative to cardiac biopsy for DCM. Second, the close association between HS and cardiac fibrosis facilitates in-depth understanding of the pathogenesis of DCM. Next, targeting overexpressed HS in DCM may be a potential way to inhibit cardiac inflammation and fibrosis. Since PI-88 has been already used to treat the malignant tumor, further investigation might enlarge the clinical application of this old drug on DCM. Moreover, classification of patients with DCM based on the heterogeneity of HS could offer a novel sight into personalized management of DCM in the future.

## Conclusion

Heparan sulfate is closely associated with immune activation, cardiac fibrosis, and heart failure in DCM. The overexpression of HS may be related to the alteration of HS metabolism. A novel molecular classification of DCM is established based on HSPGs.

## Data Availability Statement

Publicly available datasets were analyzed in this study. The original contributions presented in the study are available in the open source GEO database (https://www.ncbi.nlm.nih.gov/geo/), including GSE116250, GSE141910, GSE42955, GSE5406, and GSE121893.

## Author Contributions

LW, JC, and KH designed and supervised the study. WS and ZD analyzed the data. WS wrote the manuscript. LW, JC, FL, and LH revised the manuscript. All authors contributed to the article and approved the submitted version.

## Funding

This work was sponsored by the Shanghai Sailing Program (no. 20YF1405400), the fellowship of the China Postdoctoral Science Foundation (no. 2020M671001), the Youth Research Fund of Zhongshan Hospital, Fudan University (no. 2020ZYYS-003), the National Natural Science Foundation of China (nos. 82060094 and 81860159), the key program of Guizhou Provincial Science and Technology Fund (no. ZK-2021-003), and the Science and Technology Fund of Guizhou Health Committee (no. GZWJKJ2020-1-105).

## Conflict of Interest

The authors declare that the research was conducted in the absence of any commercial or financial relationships that could be construed as a potential conflict of interest.

## Publisher's Note

All claims expressed in this article are solely those of the authors and do not necessarily represent those of their affiliated organizations, or those of the publisher, the editors and the reviewers. Any product that may be evaluated in this article, or claim that may be made by its manufacturer, is not guaranteed or endorsed by the publisher.
