# Supplementary material for: Identification of Heparan Sulfate in Dilated Cardiomyopathy by Integrated Bioinformatics Analysis
Source: Front Cardiovasc Med. 2022 May 27;9:900428. doi: 10.3389/fcvm.2022.900428 (PMC9197211; doi:10.3389/fcvm.2022.900428)
Supplement: Supplementary file 1 [file Data_Sheet_1.docx]

Supplementary Materials

Table S1 Transcriptome data of DCM and normal heart tissues

| GEO | Platform | Tissue | Samples (Number) | | | Experiment Type | Author/Reference |
| --- | --- | --- | --- | --- | --- | --- | --- |
|  |  |  | Total | Normal | DCM |  |  |
| GSE116250 | GPL16791 | Left ventricle | 51 | 14 | 37 | high throughput sequencing | Sweet ME[1] |
| GSE141910 | GPL16791 | Left ventricle | 332 | 166 | 166 | high throughput sequencing | Margulies KB |
| GSE42955 | GPL6244 | Left ventricle | 17 | 5 | 12 | Array | Molina-Navarro MM[2] |
| GSE5406 | GPL96 | Left ventricle | 102 | 16 | 86 | Array | Hannenhalli S[3] |

**
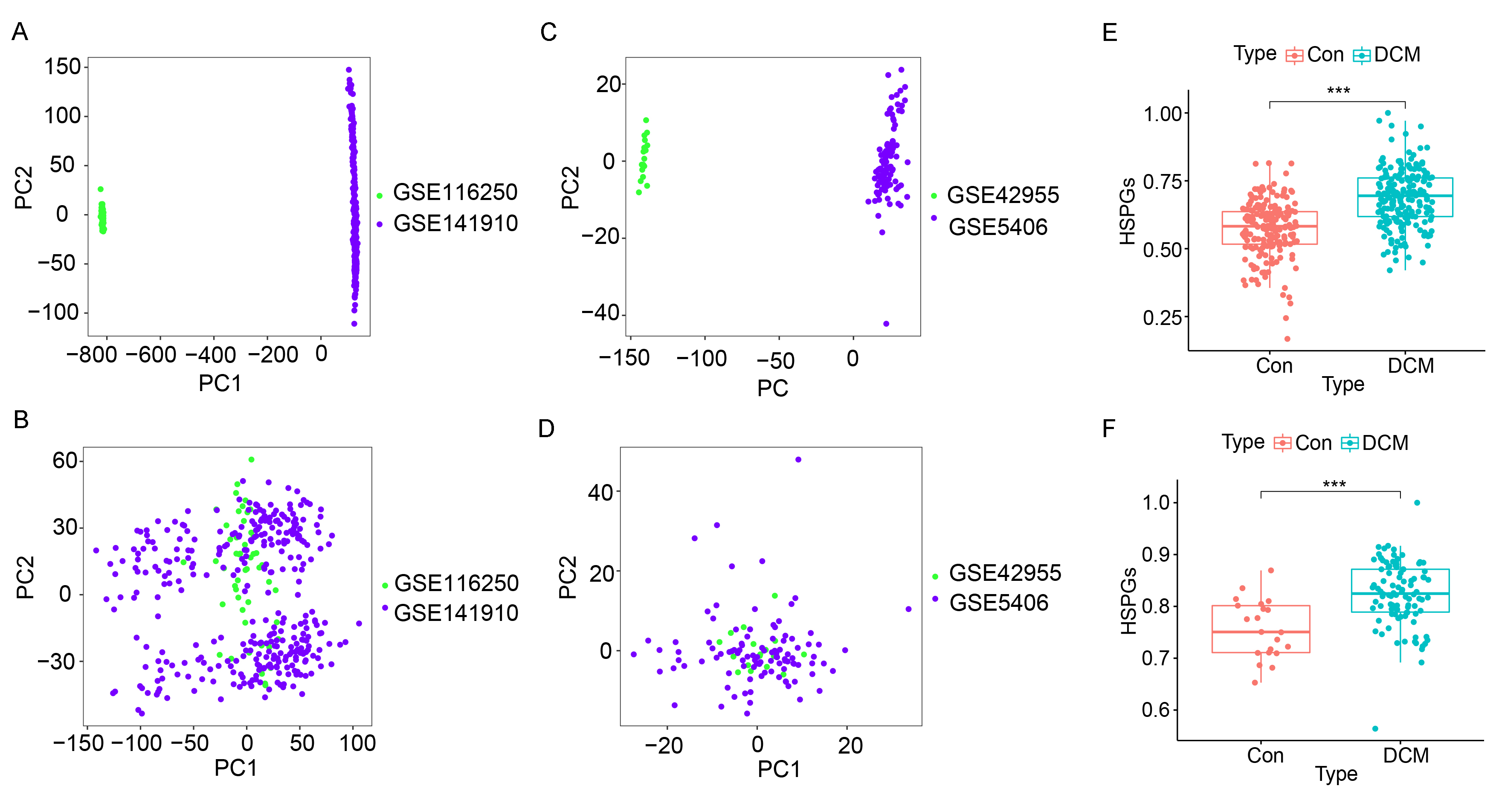
**

**Figure S1** The enrichment score of HSPGs in normal and DCM hearts. Pre (A) and post (B) batched and normalized RNA sequencing data (Training dataset). Pre (C) and post (D) batched and normalized microarray data (Validation dataset). Enrichment score of HSPGs in normal and DCM hearts calculated by (E) Training dataset and (F) validation dataset ***p<0.001.


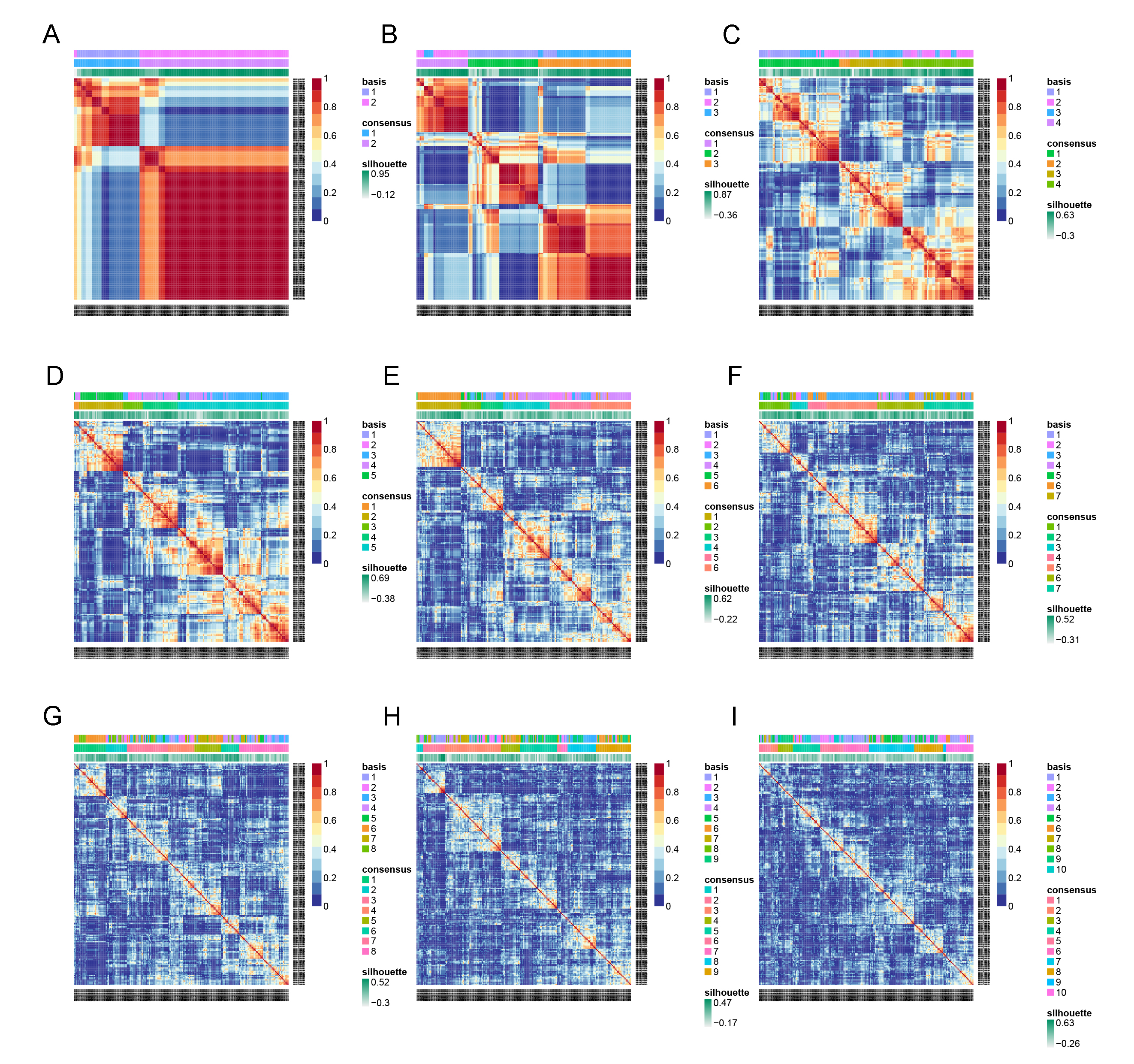


**Figure S2** The consensus matrix heatmap for k = 2-10 (A-I) was shown.


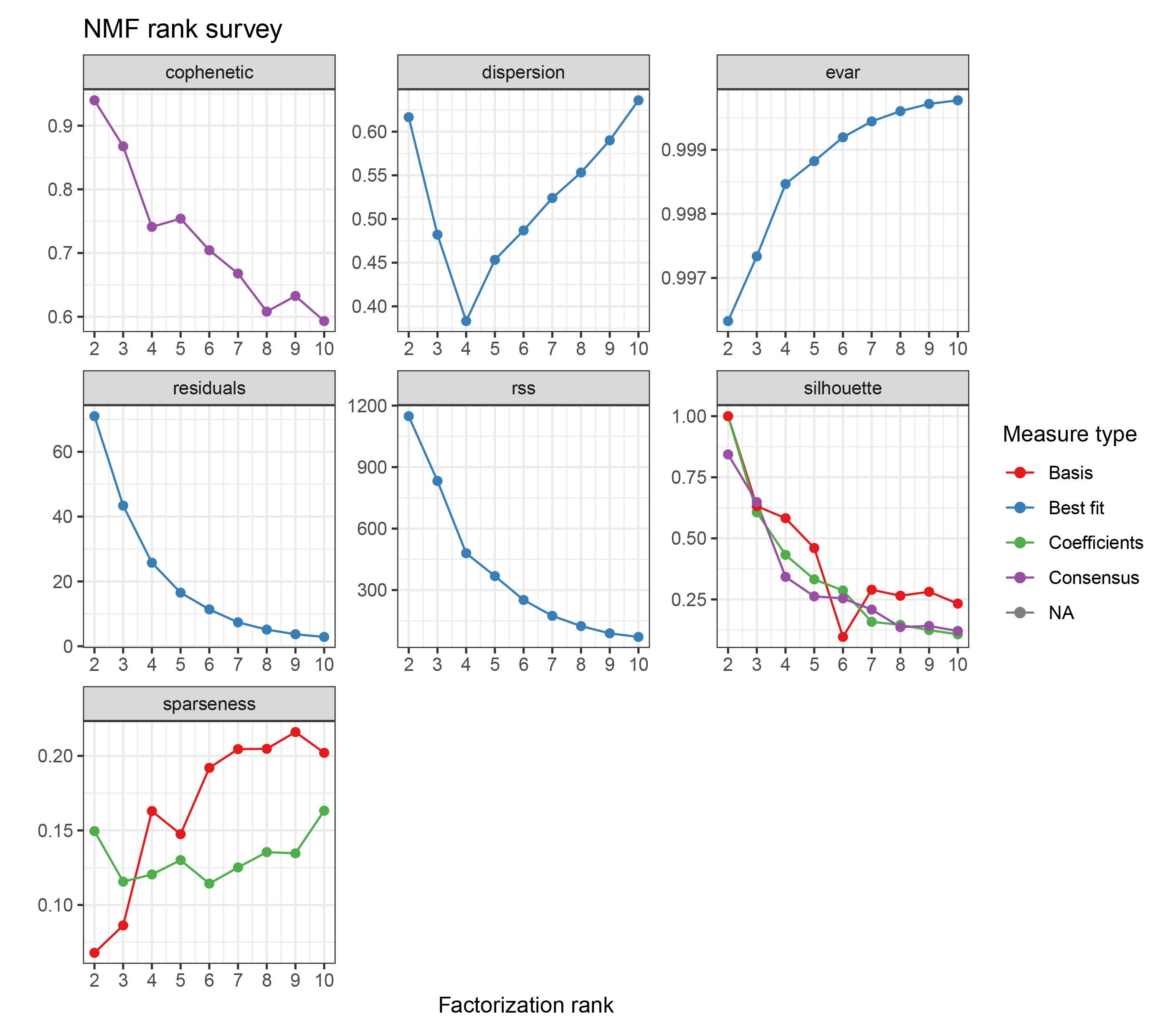


**Figure S3** NMF rank survey for k = 2-10 was shown.


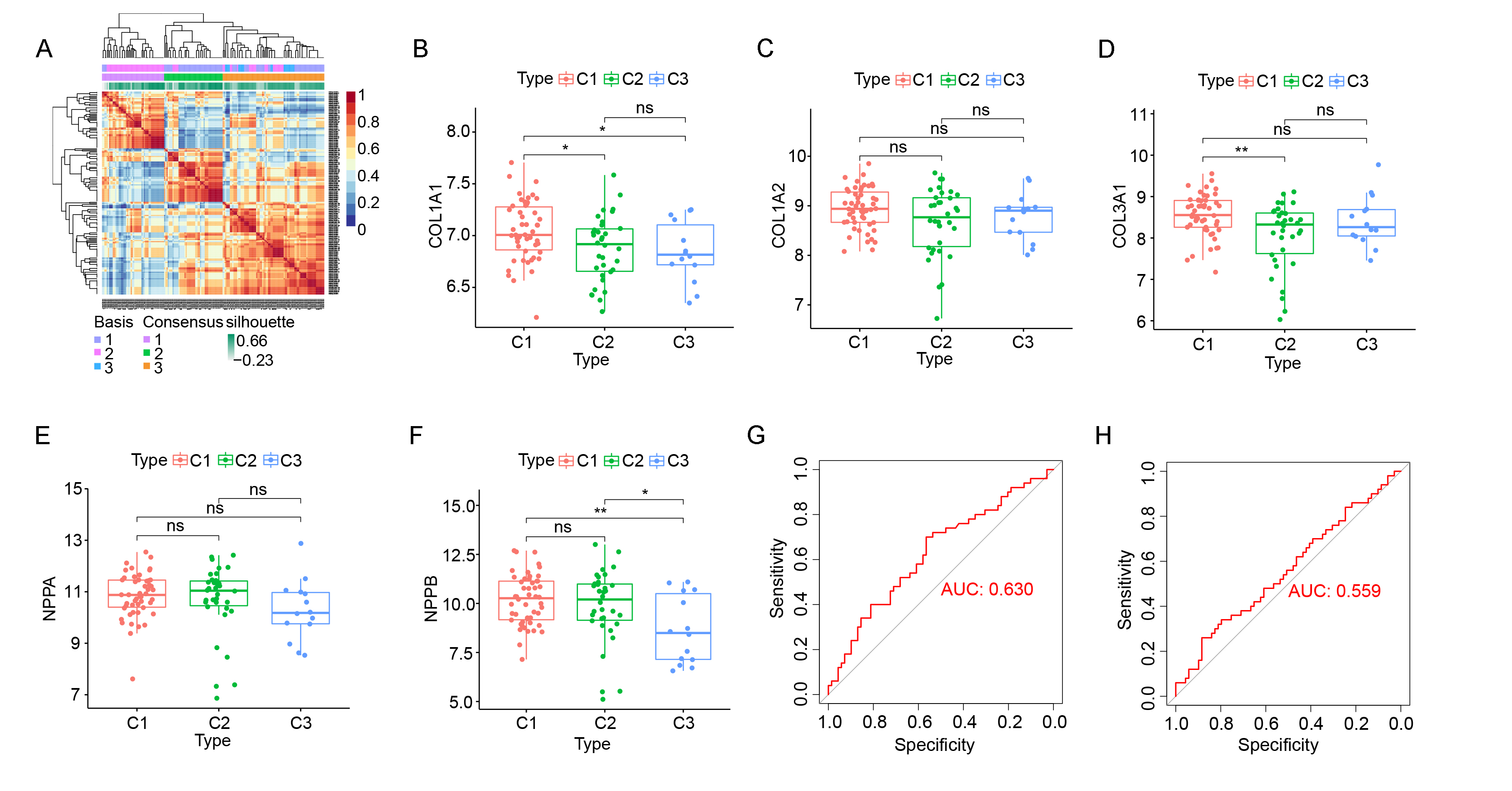


**Figure S4** Validation of molecular subtype of DCM based on HSPGs through microarray datasets. (A) NMF clustering heatmap when k = 3. (B-D) The expression of COL1A1, COL1A2 and COL3A1 among three identified subtypes. (E-F) The expression of NPPA and NPPB among three identified subtypes. (G-H) ROC curve of SDC1 expression and T cell (MCPcounter) abundance in identifying C1 subtype. *p<0.05, **p<0.01, ns = not significant.

**References**

[1] M.E. Sweet, A. Cocciolo, D. Slavov, K.L. Jones, J.R. Sweet, S.L. Graw, T.B. Reece, A.V. Ambardekar, M.R. Bristow, L. Mestroni, and M.R.G. Taylor, Transcriptome analysis of human heart failure reveals dysregulated cell adhesion in dilated cardiomyopathy and activated immune pathways in ischemic heart failure. BMC Genomics 19 (2018) 812.

[2] M.M. Molina-Navarro, E. Rosello-Lleti, A. Ortega, E. Tarazon, M. Otero, L. Martinez-Dolz, F. Lago, J.R. Gonzalez-Juanatey, F. Espana, P. Garcia-Pavia, J.A. Montero, M. Portoles, and M. Rivera, Differential gene expression of cardiac ion channels in human dilated cardiomyopathy. PLoS One 8 (2013) e79792.

[3] S. Hannenhalli, M.E. Putt, J.M. Gilmore, J. Wang, M.S. Parmacek, J.A. Epstein, E.E. Morrisey, K.B. Margulies, and T.P. Cappola, Transcriptional genomics associates FOX transcription factors with human heart failure. Circulation 114 (2006) 1269-76.
